# Entanglement is a costly life‐history stage in large whales

**DOI:** 10.1002/ece3.2615

**Published:** 2016-12-11

**Authors:** Julie van der Hoop, Peter Corkeron, Michael Moore

**Affiliations:** ^1^ Massachusetts Institute of Technology‐Woods Hole Oceanographic Institution Joint Program in Oceanography/Applied Ocean Science and EngineeringCambridgeMAUSA; ^2^ Biology DepartmentWoods Hole Oceanographic InstitutionWoods HoleMAUSA; ^3^ NOAA FisheriesNortheast Fisheries Science CenterWoods HoleMAUSA

**Keywords:** bioenergetics, blubber, capital breeder, cetacean, emergency life‐history stage, energy storage, *Eubalaena glacialis*, marine mammal

## Abstract

Individuals store energy to balance deficits in natural cycles; however, unnatural events can also lead to unbalanced energy budgets. Entanglement in fishing gear is one example of an unnatural but relatively common circumstance that imposes energetic demands of a similar order of magnitude and duration of life‐history events such as migration and pregnancy in large whales. We present two complementary bioenergetic approaches to estimate the energy associated with entanglement in North Atlantic right whales, and compare these estimates to the natural energetic life history of individual whales. Differences in measured blubber thicknesses and estimated blubber volumes between normal and entangled, emaciated whales indicate between 7.4 × 10^10^ J and 1.2 × 10^11^ J of energy are consumed during the course to death of a lethal entanglement. Increased thrust power requirements to overcome drag forces suggest that when entangled, whales require 3.95 × 10^9^ to 4.08 × 10^10^ J more energy to swim. Individuals who died from their entanglements performed significantly more work (energy expenditure × time) than those that survived; entanglement duration is therefore critical in determining whales’ survival. Significant sublethal energetic impacts also occur, especially in reproductive females. Drag from fishing gear contributes up to 8% of the 4‐year female reproductive energy budget, delaying time of energetic equilibrium (to restore energy lost by a particular entanglement) for reproduction by months to years. In certain populations, chronic entanglement in fishing gear can be viewed as a costly unnatural life‐history stage, rather than a rare or short‐term incident.

## 1 Introduction

Periods of fasting and feeding are natural for wild animals, with consequent adaptation to withstand food limitations imposed by their environment or their life histories. Energy is stored for times of deficit, and the energy budget is balanced over the long term. Migratory baleen whales go through periods of fattening and thinning, storing energy on the foraging grounds in recovery from and preparation to their depletion during migration, breeding, and lactation where individuals may be fasting and energetic costs can be high (Christiansen, Vikingsson, Rasmussen, & Lusseau, 2013; Miller, Best, Perryman, Baumgartner, & Moore, 2012).

Major changes in nutritive condition and reproduction can occur in response to good or bad prey years or environmental conditions, being natural, unforeseen circumstances (Rolland et al., 2016; Williams et al., 2013). Human‐induced factors may also contribute. Entanglement in fishing gear is now almost ubiquitous in some large whale populations. Scarring of individually identified humpback whales (*Megaptera novaeangliae*) indicates that more than a quarter of the North Pacific (29%–60%; NFWF 2007), over half the Alaskan (52%; Neilson, Straley, Gabriele, & Hills, 2009), and over three quarters of the North Atlantic (78%; Robbins, 2012) populations have been entangled at least once. Further, 8%–25% of the individuals in these populations acquire new entanglement scars every year. Many individuals in some populations have been observed carrying gear (11% of North Atlantic right whales; *Eubalaena glacialis*; Robbins, Knowlton, & Landry, 2015), and do so for months to years (van der Hoop et al., 2016). Entanglement in fishing gear is the leading cause of death for large whales in the western North Atlantic (van der Hoop, Moore, Barco, et al., 2013) and contributes mortality to marine mammal species worldwide (Clapham, Young, & Brownell, 1999; Fowler, 1987; Read, Drinker, & Northridge, 2006), but the issue also affects individual whales that survive the incident.

Entangled whales are subject to additional drag forces, likely increasing the cost of locomotion and in some cases leading to negative energy balance (Feldkamp, Costa, & DeKrey, 1988; van der Hoop, Moore, Fahlman, et al., 2013). The drag imposed by gear varies considerably based on its dimensions and configuration: Based on 15 sets of gear measured by van der Hoop et al. (2016), entanglement increases drag forces by 1.5‐fold on average, and up to 3.1‐fold in the case of a weighted lobster trap. The onset of entanglement is unpredictable, and the duration is hours to years (e.g., van der Hoop et al., 2016).

Blubber and lipid stores can be considered an energy currency with which to evaluate the energetic consequences and sublethal impacts of various stressors in cetaceans and many other mammal species (e.g., Miller et al., 2011; Williams et al., 2013). Excess energy is deposited in lipid stores which become the primary energy source during fasting (Lockyer, 1986; Worthy & Lavigne, 1987), illness (Dunkin, McLellan, Blum, & Pabst, 2010; Koopman, Pabst, McLellan, Dillaman, & Read, 2002), or increased nutritional demand (e.g., pregnancy; Miller et al., 2011). Lipid catabolism can be directly observed and measured as significant reductions in body girths and blubber thicknesses; differences in blubber thicknesses, volumes, or masses between individuals with time or under different conditions represent the amount of energy derived from stores, and can be attributed to changes in environmental conditions or particular life‐history events (Lockyer, 1981a, 1986; Worthy & Lavigne, 1987). Separately, changes in the force balance on an animal can elicit behavioral or postural responses (Feldkamp et al., 1988; van der Hoop, Moore, Fahlman, et al., 2013, 2014). Increased drag requires increased thrust or propulsive power and therefore energy expenditure (Feldkamp, 1985; Webb, 1975). As a result, chronically entangled marine animals are often emaciated (Barratclough et al., 2014; Cassoff et al., 2011). Estimates of additional drag forces and thrust power over time can indicate how much additional work is required by entangled whales compared with their nonentangled conspecifics. However, these estimates evaluate only the additional cost for locomotion, not the associated costs of stress, injury, or healing.

How much additional energy do entangled whales expend, and what are the relative costs of entanglement compared with other energetically costly life‐history events? We present two separate but complementary bioenergetic approaches to estimate the amount of additional work and energy associated with entanglement in fishing gear in a large whale species: (1) changes in blubber thicknesses and volumes between normal and entangled, emaciated whales; and (2) increased thrust power requirements to overcome measured drag forces.

The North Atlantic right whale (hereafter right whale) is a species with exceptionally high entanglement rates (83% of the population; Knowlton, Hamilton, Marx, Pettis, & Kraus, 2012; Knowlton, Hamilton, & Pettis, 2012), and where population health and reproductive rates are variable and in recent decline (Fujiwara & Caswell, 2001; Pettis et al., 2004; Robbins et al., 2015; Rolland et al., 2016). Long‐term individual sightings, life history, and health data are available, as is information on entanglements, including the gear involved. We use observations, measurements, theory, and available literature to outline the energetic life history of right whales in particular, to contextualize the demands, time course, and extent of entanglement in fishing gear.

## 2 Methods

### 2.1 Changes in blubber thickness and volume

We obtained dorsal axillary blubber thicknesses and body lengths of dead right whales measured at necropsy from the North Atlantic Right Whale Consortium (NARWC) necropsy database (NARWC 2015). We assumed that individuals that were not described as emaciated and whose cause of death was not related to entanglement were in normal body condition. Because adults for which blubber thicknesses were available had significantly greater body lengths (1418 ± 87 cm) than juveniles (1097 ± 129 cm; one‐way ANOVA *F*_1,15_ = 32.788, *p *<* *.0001), they also had significantly larger estimated body widths. We therefore separated individuals into two life stages for statistical analysis. We have provided a list of symbols and abbreviations for reference (Table 1).

**Table 1:** List of symbols and abbreviations

Symbol	Definition	Unit
*a*	Ellipsoid major axis	m
*b*	Ellipsoid minor axis	m
*Ba*	Basal cost	
*Br*	Breeding cost	
*c*	Ellipsoid minor axis	m
*d*	Entanglement duration	s
*D*	Drag force	N
*Fr*	Foraging cost	
*Fd*	Fat deposition cost	
η	Overall efficiency	
η_*m*_	Metabolic efficiency	
η_*p*_	Propulsive efficiency	
*l*	Body length	cm
*L*	Lactation cost	
*M*	Migration cost	
*P*	Pregnancy cost	
*P*_*T*_	Thrust power	W
ρ	Density	kg/m^3^
*T*	Measured blubber thickness	cm
*U*	Speed	m/s
*V*	Total body volume	m^3^
*W*_a_	Additional work	J

**Figure 1:**
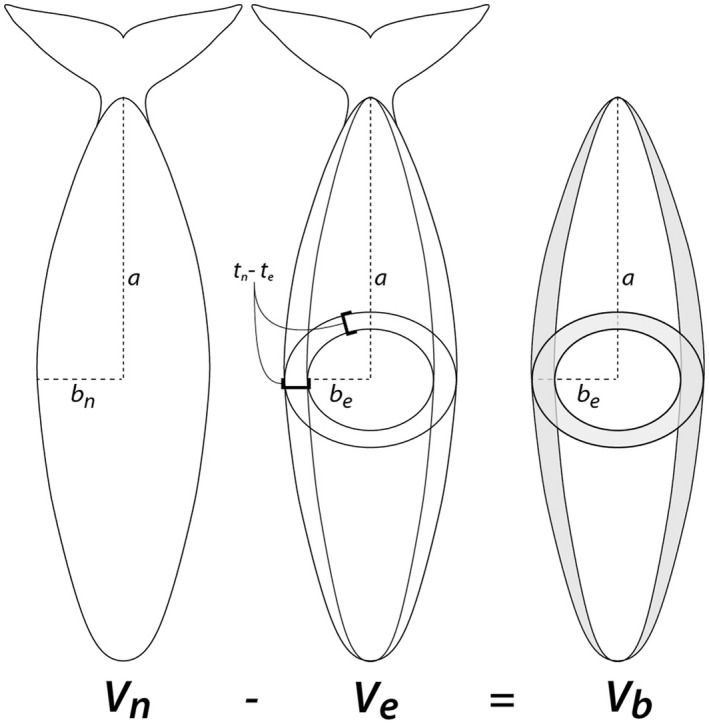
Schematic representing blubber volume estimation (*V*_*b*_) for a North Atlantic right whale of body length *a *×* *2. Total body volume of normal whales (*V*_*n*_) was estimated from length and body radius *b*_*n*_ = *c*_*n*._ Body volumes of entangled whales (*V*_*e*_) were based on length and body radius *b*_*e*_, based on the difference in blubber thicknesses between normal (*t*_*n*_) and entangled (*t*_*e*_) individuals measured at necropsy. See text for detail

We estimated total body volumes (*V*; m^3^) for each individual by approximating whales as rotationally symmetric ellipsoids (Figure 1), with major axis *a *= half the body length (*l*; cm), and minor axes *b*_*n*_ = *c*_*n*_ = half the body width (*w*; cm) as in Klansjcek, Nisbet, Caswell, and Neubert (2007). We calculated body widths for whales in normal body condition as in Fortune et al. (2012) and reduced body widths of entangled animals (*b*_*e*_ = *c*_*e*_) by the difference in measured blubber thicknesses between normal (*t*_*n*_) and entangled (*t*_*e*_) whales:

(1) $$\begin{array}{cc} & b_{e}=b_{n}-\left(t_{n}-t_{e}\right)\\ & c_{e}=c_{n}-\left(t_{n}-t_{e}\right) \end{array}$$

We calculated total body volumes of each whale based on normal body proportions (*V*_*n*_) and with thinner blubber layers (*V*_*e*_, using *b*_*e*_ and *c*_*e*_), as:

(2) $$V=\frac{4}{3}\pi abc.$$

We assumed the difference between the normal and entangled body volumes of each individual (*V*_*n*_ − *V*_*e*_) represents the volume of blubber catabolized between the two conditions (Figure 1).

We converted blubber volumes to energy (J) based on published values of lipid content (61.8%; Montie et al., 2010), blubber density (900 kg/m^3^; Parry, 1949), caloric content of lipid (9450 kcal/kg), and energy (4184 J/kcal; Lockyer, 1978; Schmidt‐Nielsen, 1997). Because lipid content measurements can vary with sampling method (Krahn et al., 2004; Woodley, Brown, Kraus, & Gaskin, 1991), blubber depth, and body location (Koopman et al., 2002; Struntz et al., 2004), we established high and low estimates of 61.8 ± 25% and assumed constant lipid content and density. We assume no difference in lipid density between normal and entangled individuals and calculate blubber lipid metabolism only (i.e., not considering energy from protein catabolism or other body lipid reserves; see Discussion). We used two‐way ANOVA to determine whether blubber thicknesses were significantly different between life stages (juvenile, adult) or condition (normal, entangled).

### 2.2 Increased power requirements

To overcome the increased drag forces associated with fishing gear, entangled whales increase power for propulsion (thrust power, *P*_*T*_; Watts). Over time, this requires additional work (*W*_a_; J) and therefore energy consumed (J) by the animal. We calculated thrust power from measured drag forces (*D*; N) for 15 right whales, whose entangling gear was measured with a tensiometer (van der Hoop et al., 2016) and estimates of swimming efficiency (η) specific to the entangled (subscript *e*;* P*_*T*,*e*_, *D*_*e*_, η_*e*_) and nonentangled (subscript *n*;* P*_*T*,*n*_, *D*_*n*_, η_*n*_) conditions:

(3) $$P_{T,e}=\frac{D_{e}U_{e}}{\eta_{e}},\;P_{n}=\frac{D_{n}U_{n}}{\eta_{n}}.$$

Swimming efficiency is a combination of propulsive efficiency (η_*p*_) and muscular efficiency (η_*m*_ = 0.25). We based values of swimming efficiency on the maximum propulsive efficiencies calculated from bio‐logging tag data for right whale EG 3911 averaged over dive descents and ascents when entangled and following disentanglement (recorded 15 January 2011; van der Hoop, Moore, Fahlman, et al., 2013; van der Hoop et al., In Press ESR):

(4) $$\begin{array}{cc} & \eta_{e}=\eta_{p}\times \eta_{m}=0.50\times 0.25=0.13\\ & \eta_{n}=\eta_{p}\times \eta_{m}=0.51\times 0.25=0.13. \end{array}$$

This individual had been entangled in at least 72 m of gear (~93 N; van der Hoop et al., In Press ESR) for months and showed significantly compromised health and body condition. We therefore applied maximum propulsive efficiencies, which provide a conservative estimate. We assumed a constant‐velocity scenario (*U*_*e*_ = *U*_*n*_), *that is*, that animals do not slow down once entangled as few data exist or show consistent changes in speed with entanglement (see van der Hoop et al., In Press ESR).

We calculated the additional thrust power (*P*_*T*,*a*_; W) as the difference in propulsive power between the entangled and nonentangled conditions, and the additional work (*W*_*a*_; J) required for propulsion by an entangled whale as this additional power sustained over the minimum and maximum durations (seconds; *d*_min_ and *d*_max_, respectively) of each entanglement:

(5) $$W_{a,\min}=d_{\min}\left(P_{T,e}-P_{T,n}\right),$$

(6) $$W_{a,\max}=d_{\max}\left(P_{T,e}-P_{T,n}\right).$$

We built entanglement timelines and associated increases in drag, power, and work from sightings records and disentanglement histories for all whales (Figure S1). We calculated maximum entanglement durations (*d*_max_) from the last gear‐free sighting before entanglement and either the first gear‐free sighting following disentanglement, confirmed death (carcass detection and identification), or presumed death (once an individual has not been sighted in 6 years; Knowlton, Kraus, & Kenney, 1994). We calculated minimum entanglement durations (*d*_min_) from the first entangled sighting and either the date (1) of disentanglement (including partial disentanglement), (2) last seen entangled, or (3) that an attached telemetry buoy ceased transmissions. At the onset of entanglement, we estimated increased power from entangled drag measurements (*D*_*e*_) from van der Hoop et al. (2016). We incorporated information from each whale's history to reflect changes in drag from disentanglement response. We included drag from adding the satellite telemetry buoy based on measured values from van der Hoop et al. (2016). We reduced the total drag of entangled animals (*D*_*e*_) in the event of disentanglement attempts that were successful in reducing the length of trailing line or removing floats; we calculated the change in expected mean drag from the linear relationship in van der Hoop et al. (2016) with dimensions of the original and altered entangling gear. We ignored drag added for single‐day disentanglement events (e.g., to slow the animal through a process known as *kegging*) as (1) these details were not consistently recorded, (2) the drag of these buoys used were never measured, and (3) these events occur over short (<12 hr) durations.

We sought to determine whether the fate of each whale was related to the additional power output (*P*_*T*,*a*_), or minimum or maximum additional work (*W*_a,min_, *W*_a,max_) associated with its entanglement. To do so, we compared these variables for individuals who died vs. survived their entanglements with paired *t* tests. We defined a critical level of minimum additional work associated with entanglement‐related mortality as the 0.75 quantile of the minimum additional work performed by whales that did not survive (i.e., of *W*_a,min_(fate = dead)).

### 2.3 Life‐history context

**Figure 2:**
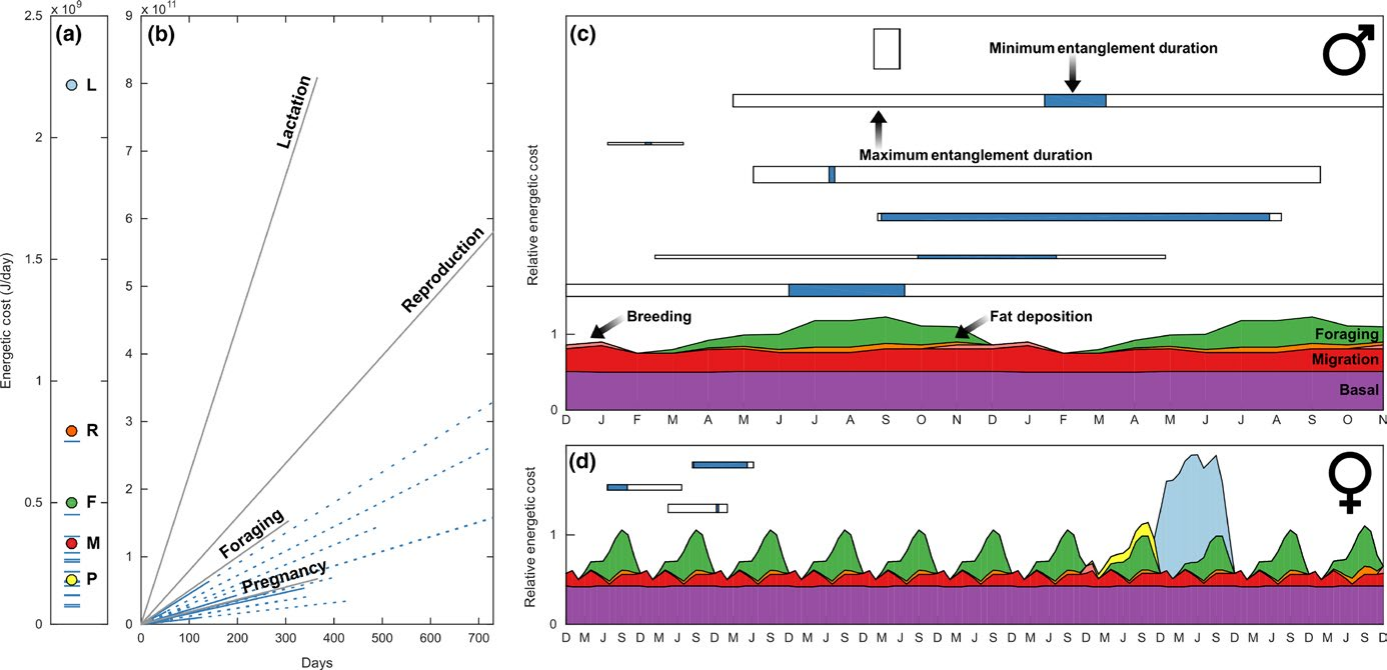
(a) Fixed‐rate daily energetic costs (J/day) from drag associated with entanglement in fishing gear (blue lines) compared with costs of other life‐history events: pregnancy (yellow, *P*; Fortune et al., 2013), migration (red, *M*; van der Hoop, Moore, Fahlman, et al., 2013), foraging (green. *F*; McGregor, 2010), reproduction (orange, *R*; pregnancy + lactation; Klansjcek et al., 2007), and lactation (light blue, *L*; Fortune et al., 2013). (b) Fixed‐rate daily energetic costs projected over the duration of each event or season. For entanglements, solid lines show the minimum entanglement duration and dashed lines the maximum entanglement duration. (c and d) Relative energetic timelines of males and females show cyclic energy demands in migratory whales. Horizontal bars show the temporal onset and extent of the minimum (blue) and maximum (white) entanglement durations. The timing of three female entanglements is shown relative to their known calving events; five females in the study but not in this figure have unknown calving histories. The thickness of each bar indicates the cost of each entanglement relative to other life‐history events (*n *=* *7 males, *n *=* *3 females). The vertical placement of each bar simply distinguishes individuals

To put the energetic demands of entanglement in context, we compared the estimated cost per day and duration of other right whale life‐history events from the literature (Figure 2). For example, van der Hoop, Moore, Fahlman, et al. (2013) estimate a one‐way, 22‐day migration costs 7.3 × 10^9^ J, or 3.3 × 10^8^ J/day. Foraging requires energy for diving and searching, and to counter the increased drag associated with filter feeding—approximately 5 × 10^8^ J/day (McGregor, 2010; Simon, Johnson, Tyack, & Madsen, 2009). While Klansjcek et al. (2007) estimated combined 2‐year reproductive costs of pregnancy and lactation at 7.9 × 10^8^ J/day, Fortune, Trites, Mayo, Rosen, and Hamilton (2013) estimated the costs of pregnancy and lactation separately: The difference between daily energetic requirements of resting females (1.9 × 10^9^ J/day) and of pregnant (2.1 × 10^9^ J/day) and lactating females (4.1 × 10^9^ J/day) suggests daily costs of pregnancy and lactation are 1.8 × 10^8^ J/day and 2.2 × 10^9^ J/day, respectively. We projected daily energy costs of life‐history events over their duration to provide a comparison of daily additional energetic costs to entangled whales over the minimum and maximum durations of their entanglements.

We also represented seasonal variability in these costs and compared their relative contributions to individual energy budgets for nonentangled adult male and female right whales. Above basal (*Ba*) costs, annual migration (*M*), foraging (*Fr*), and breeding (*Br*) contribute to varying energetic demands including periods of nutritional excess (hyperphagia) and deficit (hypophagia; McNamara & Houston, 2008). Pregnancy (*P*) and lactation (*L*) add especially costly demands in females. We represented the annual cost of living for a right whale following, for example, Gessaman (1973) and West (1960), by estimating monthly relative costs of life‐history events through the year separately for males (Equation (7)) and females (Equation (8)):

(7) $$\begin{array}{cc} & \text{Male Relative Energy Budget}\;\\ & \left(\text{MEB}\right)\\ & =\sum_{m=1}^{12}Ba_{m}+M_{m}+Fr_{m}+Fd_{m}+Br_{m}\cdot \frac{\text{MEB}}{12}=1 \end{array}$$

(8) $$\begin{array}{cc} & \text{Female Relative Energy Budget}\;\\ & \left(\text{FEB}\right)\\ & =\sum_{m=1}^{48}Ba_{m}+M_{m}+Fr_{m}+Fd_{m}+Br_{m}+P_{m}+L_{m}\cdot \frac{\text{FEB}}{48}=1 \end{array}$$

For a male, energy equilibrium is reached after a full annual cycle. For females, equilibrium is reached after 4 years: pregnancy, lactation, and a 2‐year resting period, reflecting the mean calving interval in the right whale population in the recent past (Knowlton, Hamilton, & Pettis, 2012; Knowlton, Kraus, & Kenney, 1994). We then calculated relative additional costs due to entanglement and added these costs to the budget.

We assumed basal existence energy costs (*Ba*; for thermoregulation, digestion, and excretion) as ~50% of the budget, remaining fairly continuous throughout the year; there is little evidence for seasonal resting metabolic rate fluctuations in cetaceans (Rechsteiner, Rosen, & Trites, 2013). We constrained the schematic to have a maximum sustained energy of 2.4× and 4.5× basal costs for males and females, respectively, on the conservative end of the maximum sustained energy of 4–7× basal metabolic rates across species (Speakman & Krol, 2010; Weiner, 1992).

We estimated migration costs (*M*) from the relative movement of right whales between habitats (Brillant, Vanderlaan, Rangeley, & Taggart, 2015; Schick et al., 2013; Vanderlaan, 2010), where movement is greatest in January–February and April–May. Males and females have similar overall movement patterns, although destinations and transition probabilities between habitats can differ (Schick et al., 2013). To reflect residence times on the calving grounds, we reduced movement costs in February–March (Fortune et al., 2013; Schick et al., 2013) and in feeding areas in June–August (Vanderlaan, 2010). Although monthly residence probabilities can be high for some habitats (e.g., 66% in the Bay of Fundy; Vanderlaan, 2010), individual whales can be highly transitory between north temperate habitats on shorter timescales (Brillant et al., 2015; Mate, Nieukirk, & Kraus, 1997).

Right whales forage seasonally on Calanoid copepods in surface waters (e.g., in Cape Cod Bay; (Mayo & Marx, 1990; Parks, Warren, Stamieszkin, Mayo, & Wiley, 2012)) and at depth (e.g., in the Bay of Fundy, Roseway Basin); foraging costs (*Fr*) increase to a maximum in July–September, when foraging rates are greatest (Baumgartner & Mate, 2003).

On the feeding grounds, large amounts of energy are stored in lipid reserves (Christiansen et al., 2013; Miller et al., 2011). Fat deposition is efficient, roughly 5%–15% per kilocalorie of metabolizable energy; protein deposition costs can be much higher (2.25–2.38 kJ/kJ deposited; Pullar & Webster, 1977; van Es, 1977; Roberts & Young, 1988). Costs of fat deposition are therefore related to energy acquisition, a function of foraging costs (i.e., effort). Based on previous budgets (Gessaman, 1973; West, 1960), we assume fat deposition (Fd) to be 2% of foraging costs. Costs of breeding (*Br*) are incurred for mate searching and social displays (Kraus & Hatch, 2001).

We included breeding costs only prior to pregnancy for females. Pregnancy costs 1.09× as much as the nonreproductive female (Fortune et al., 2013); fetal development is minimally costly and abdominal distension increases body drag only 3%–4% (McGregor, 2010). Lactation is the most energetically expensive life‐history event, costing 2.17× the nonreproductive female budget (Fortune et al., 2013). We assume weaning lasts 12 months (Hamilton & Cooper, 2010). Pregnant and lactating females have reduced fat deposition (*Fd* = 1%) and slightly reduced foraging effort as has been suggested for humpback (Szabo & Duffus, 2008) and southern right (Taber & Thomas, 1982; Thomas & Taber, 1984) whales.

We converted the additional energy from entanglement over the course of 1 day (*W*_a,day_, *e.g*., Equation (5)) for each case to relative additional energetic costs (*W*_a,rel_) by comparing them to the daily (subscript day) and relative (subscript rel) costs of foraging (*F*) and migration (*M*):

(9) $$W_{a,\mathrm{rel}}=\frac{\left(W_{a,\mathrm{day}}/F_{\mathrm{day}}\right)\times F_{\mathrm{rel}}+\left(W_{a,\mathrm{day}}/M_{\mathrm{day}}\right)\times M_{\mathrm{rel}}}{2}$$

We added these relative, monthly costs to the budget over the minimum and maximum durations of each entanglement case to obtain an entangled energy budget (MEB_*E*_ = MEB + *W*_a,rel_; FEB_*E*_ = FEB + *W*_a,rel_). We calculated the contribution of pregnancy, lactation, and maintenance to the 4‐year breeding cycle budget, to which we compared entanglement costs over minimum and maximum durations; we then calculated the additional energetic demand of entanglement on top of the total 4‐year budget. Assuming finite entanglements, we calculated the time for return to energetic equilibrium (for entanglement costs to be recouped and for recovery in preparation for the next pregnancy) as the surplus energy required for entanglement divided by the monthly energy available in the absence of reproduction.

## 3 Results

### 3.1 Blubber thickness

**Figure 3:**
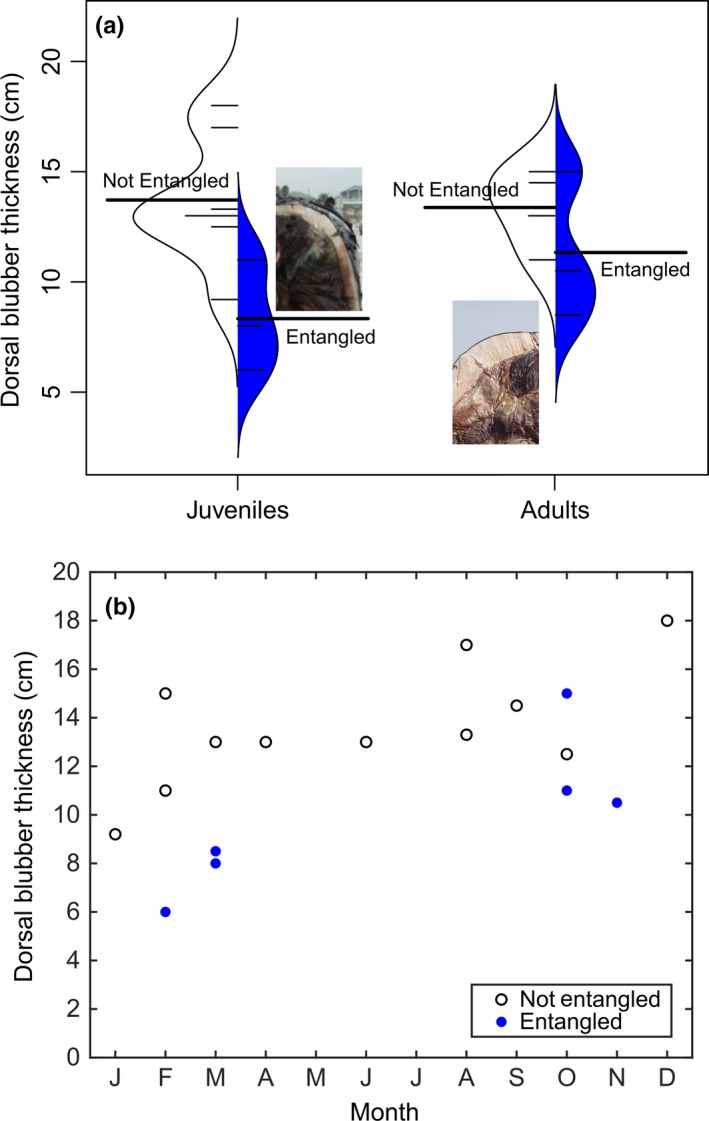
Dorsal axillary blubber thickness (cm) measured at necropsy in juvenile and adult North Atlantic right whales whose cause of death was not related to entanglement (white) or due to entanglement (blue). (a) Curved vertical lines show the distributions of each subgroup; short horizontal lines are individual samples, thick horizontal lines are population means (Kampstra, 2008). Inset photographs are equally scaled and show blubber of an entangled juvenile (EG 3911) and a nonentangled adult (EG 1004). (b) Dorsal blubber thicknesses by month of death

Entangled juveniles (*n *=* *3, 8.3 ± 2.5 cm) and adults (*n *=* *3, 11.3 ± 3.3 cm) had significantly thinner dorsal blubber layers (*t*_*e*_) at necropsy compared with nonentangled individuals in the same life stages (*t*_*n*_; juveniles *n *=* *7, 13.8 ± 2.7 cm; adults *n *=* *4, 13.4 ± 1.8 cm; two‐way ANOVA, *F*_1,14_ = 7.16, *p *=* *.018; Figure 3a; Table 2). Whales that died from entanglement had thinner blubber layers, even considering seasonality in blubber stores (Figure 3b).

**Table 2:** New England Aquarium Catalog and Field identification number, age, sex, length (cm), weight (kg), date of necropsy, and measured dorsal blubber thickness of juvenile and adult North Atlantic right whales that died as a result of entanglement or due to other causes

		Catalog Number	Field Number	Age	Sex	Length (cm)	Date of Necropsy	Dorsal Blubber Thickness (cm)
Juveniles	Other causes		NY‐2680‐2001		F	910	19 June 2001	13.0
		3710	CALO 0901		M	975	26 January 2009	9.2
		1504	MH86‐142‐Eg		F	1090	7 August 1986	17.0
			JGM 415504886MH‐76‐056‐Eg	3	M	1100	5 March 1979	13.0
		2450		min 2	F	1259	21 August 1997	13.3
		3508	GA2006025Eg	2	M	1260	30 December 2006	18.0
		2250		min 3	M	1266	20 October 1995	12.5
	Entangled	3911	EgNEFL1103	2	F	1000	2 February 2011	6.0
		1907	RKB‐1420 MH91‐762‐Eg	2	F	1005	13 March 1991	8.0
		3107	MH02‐726‐Eg	1	F	1100	13 October 2002	11.0
Adults	Other causes	1223		min 12	F	1360	13 September 1992	14.5
		1014	MH99‐601‐Eg	min 28	F	1370	21 April 1999	13.0
		1623	RKB‐1429	min 12	M	1415	1 February 1996	15.0
		1004	VMSM 2004‐1004	30		1600	11 February 2004	11.0
	Entangled	2030	CCSN99‐143	min 10	F	1350	21 October 1999	15.0
		2301	VAQS‐2005‐1008Eg	12	F	1380	4 March 2003	8.5
		1238		min 19	M	1455	4 November 2001	10.5

Based on average body lengths of adults (1418 ± 87 cm) and juveniles (1097 ± 129 cm), the difference in blubber volumes between the entangled and nonentangled conditions was 3.4 and 5.4 m^3^ in these two age classes, respectively. These volume losses suggest 7.4(4.4–10.3)×10^10^ J and 1.2(0.7–1.7)×10^11^ J of energy were consumed during the course to death of lethal entanglements in adults and juveniles, respectively.

### 3.2 Power estimates

**Figure 4:**
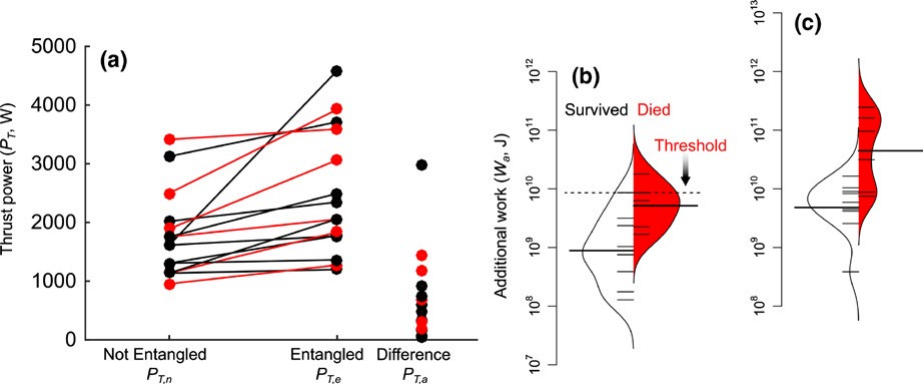
(a) Thrust power (W) of right whales swimming at 1.23 m/s when not entangled (*P*_*T*,*n*_) and when entangled in fishing gear (*P*_*T*,*e*_), as well as the additional thrust power (*P*_*T*,*a*_ = *P*_*T*,*e*_ − *P*_*T*,*n*_) required when entangled. Red dots represent whales that died from their entanglement; black represents cases that survived. (b and c) Additional work (*W*_a_, J) required for entangled right whales over their minimum (b) and maximum (c) entanglement durations, separated for whales who survived their entanglements (white) and those who died (red). The dashed line in panel B indicates the 0.75 quantile “critical additional energy” threshold used to estimate survival

Based on the histories of 15 right whale entanglements for which gear drag was measured, entanglements increased propulsive power requirements 1.48 ± 0.52‐fold (range 1.04‐ to 4.45‐fold; Figure 4a) if swimming speed was maintained; these entanglements were sustained 92 ± 101 (range 1–332) to 810 ± 1044 (range 23–3328) days (Table 3). Mean(±*SD*) daily energetic costs were 2.13(±0.92)×10^8^ J/day and ranged 1.03 × 10^8^ − 3.96 × 10^8^ J/day (Figure 2a). Over the duration of their entanglements, individuals required 3.95(±4.84)×10^9^ to 4.08(±7.19)×10^10^ J more energy than nonentangled whales to complete the additional propulsive work to overcome entanglement drag forces (Figure 4b). Individuals who died from entanglements had significantly higher minimum and maximum additional energy expenditures over their entanglement durations compared with those that survived (Table 4). The 0.75 quantile of minimum additional work performed by whales that did not survive their entanglements was 8.57 × 10^9^ J. There was no detectable difference in the increases in propulsive power associated with the entanglement configurations of cases that died vs. those that survived their entanglements (Figure 4a; Table 4).

**Table 3:** Details of fishing gear entanglements whose drag forces were measured (*n *=* *15). Sightings data provided the minimum and maximum entanglement periods (days), individual fate (S = survived; D = died), and age at entanglement (years) for all whales, from which we estimated length and mass from Moore et al. (2004)

Catalog Number	Age at entanglement	Length (cm)	Mass (kg)	Entanglement duration (days)		Fold increase in drag	Floats	Nonentangled power (*P*_*T*,*n*_, W)	Entangled power (*P*_*T*,*e*_, W)	Additional power (*P*_*T*,*a*_, W)	Additional work (*W*_a_; J)		Fate
				Min	Max						Min	Max	
EG 1102	21	1435	40,416	100	3328	1.13	0	3413	3590	176	6.28 × 10^9^	1.61 × 10^11^	D
EG 1427	18	1413	35,095	5	487	1.27	0	3126	3713	586	3.88 × 10^8^	1.04 × 10^10^	S
EG 2030	12	1357	24,453	163	769	1.68	1	2497	3926	1429	1.80 × 10^10^	9.61 × 10^10^	D
EG 2212	5	1235	1,2038	1	23	3.05	1	1614	4587	2973	1.28 × 10^8^	5.91 × 10^9^	S
EG 2212	6	1260	1,3811	332	346	1.25	0	1755	2054	299	8.57 × 10^9^	8.76 × 10^9^	D
EG 2223	8	1300	17,359	263	300	1.24	0	2021	2346	325	8.61 × 10^9^	9.01 × 10^9^	S
EG 2710	3	1164	8,490	68	397	1.46	0	1306	1776	470	3.16 × 10^9^	4.46 × 10^9^	S
EG 3107	1	1011	4,943	57	297	1.43	1	954	1273	319	1.68 × 10^9^	7.47 × 10^9^	D
EG 3294	6	1260	13,811	11	293	1.52	0	1755	2493	737	7.50 × 10^8^	1.65 × 10^10^	S
EG 3311	7	1282	15,585	51	2510	1.74	0	1890	3063	1173	5.17 × 10^9^	2.46 × 10^11^	D
EG 3314	2	1108	6,717	25	98	1.94	1	1136	2059	923	2.36 × 10^9^	8.35 × 10^9^	S
EG 3420	5	1235	12,038	12	352	1.17	0	1614	1769	155	7.60 × 10^8^	4.16 × 10^9^	S
EG 3445	2	1108	6,717	192	2459	1.73	1	1136	1834	697	2.25 × 10^9^	3.14 × 10^10^	D
EG 3610	3	1164	8,490	119	435	1.12	0	1306	1363	58	1.04 × 10^9^	2.56 × 10^9^	S
EG 3714	2	1108	6,717	5	64	1.13	0	1136	1196	60	1.76 × 10^8^	3.86 × 10^8^	S

**Table 4:** Minimum and maximum additional energy expenditures (*W*_a,min_, *W*_a,max_; J) and thrust power output (*P*_*T*_, W) of entangled North Atlantic right whales that died or survived their entanglements. *t* and *p* statistics are presented for two‐sample *t* tests

	Died (*n *=* *6)	Survived (*n *=* *9)	*t*_*13*_	*p*
Minimum additional energy (*W*_a,min_; J)	6.99 ± 5.96 × 10^9^	1.93 ± 2.71 × 10^9^	−2.25	.0424
Maximum additional energy (*W*_a,max_; J)	9.18 ± 9.62 × 10^10^	6.86 ± 4.8310^9^	−2.70	.0183
Additional power (*P*_*T*_; W)	682 ± 516	699 ± 904	0.040	.9687

### 3.3 Life‐history context

The additional energetic costs of drag from fishing gear entanglement ranged 7.24 × 10^7^ − 7.52 × 10^8^ J/day (Figure 2a). These costs exceeded daily costs of pregnancy, are comparable to those of migration and foraging, and are 3%–34% as much as daily costs of lactation (Figure 2a,b). The time of onset of entanglements is unpredictable and occurs throughout seasonal and reproductive cycles; as such, their costs can be incurred during times of high or low energy demand or availability (Figure 2c,d).

**Figure 5:**
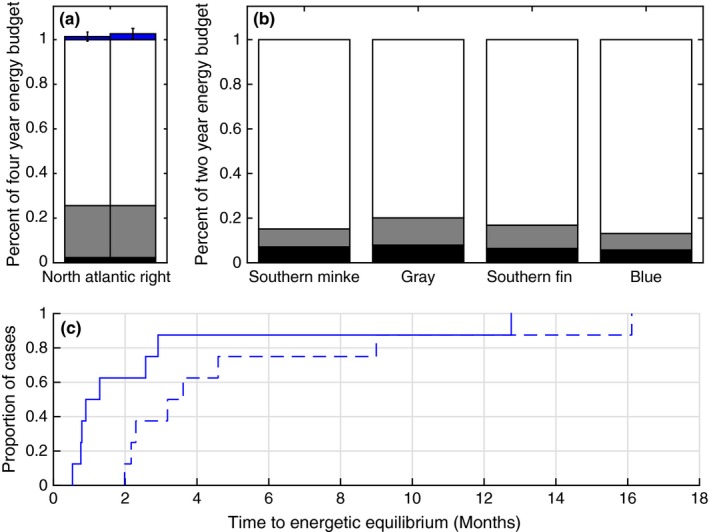
(a) Percentage of female North Atlantic right whale 4‐year energy budgets allocated to pregnancy (black), lactation (gray), and female maintenance (white) costs and mean additional costs associated with drag from entanglement in fishing gear (blue) over minimum (left) and maximum (right) entanglement durations for eight females. Error bars show *SD*. (b) Percentage of 2‐year energy budget allocated to pregnancy (black), lactation (gray), and female maintenance (white) for Southern minke (Lockyer, 1981a), gray (Villegas‐Amtmann et al., 2015), Southern fin (Lockyer, 1981b, 1987), and blue (Lockyer, 1981b) whales. (c) cumulative distribution function (CDF) of the time to energetic equilibrium (to restore energy lost by a particular entanglement) for entangled female right whales (*n *=* *8) based on their minimum (blue solid) and maximum (blue dashed) entanglement durations

For females, entanglement costs were dwarfed by the high energetic demands of lactation (Figure 2d). We estimated that female right whales spend 2% of their energy budget during a 4‐year reproductive cycle on pregnancy and 23% on lactation; 75% is allocated to maintenance and recovery of energetic stores (Figure 5a). The drag from entanglements we measured required 0.3%–6.2% to 1.0%–7.8% of the 4‐year female energy budget based on minimum and maximum entanglement durations, respectively. The median time to energetic equilibrium, to restore energy lost by a particular entanglement, is 1.0–3.3 months (minimum–maximum entanglement durations), with one case requiring 12.5–15.8 months (Figure 5c). Assuming no change in energy availability or individual behavior to compensate for energetic allocation, these added costs likely would not affect calving intervals for five females (63% of cases), requiring a minimum of <2 months to reach equilibrium, but may increase calving intervals by 1 year for three females (37% of cases) who require between 2 and 16 months (Figure 5c).

## 4 Discussion

Chronic entanglement in fishing gear affects marine animal populations worldwide (Clapham et al., 1999; Fowler, 1987; Read et al., 2006; Wegner & Cartamil, 2012). We used two approaches that yield comparable estimates of the energetics associated with increased drag from entanglement. These, in combination with individual history data, have allowed us to show that:

1. Propulsive requirements increase on average 1.58‐fold, which over time affects individual survival (Figure 4).
2. Entanglement results in the consumption of endogenous lipid energy reserves on the order of magnitude as is consumed during lactation (Figure 3).
3. Entanglement can lead to energetic demands similar in magnitude and duration to other life‐history events (Figure 2a,b).
4. The onset and duration of these entanglements have significant impacts on the energy budget of individuals (Figure 2), especially reproductive females.
5. Even short‐term or low‐drag entanglements can result in extended calving intervals in a *k*‐strategist (Figure 5c).

### 4.1 Entanglement cost and survival

Emaciation is a common observation in marine animals chronically entangled in fishing gear (Barco, D'Eri, Woodward, Winn, & Rotstein, 2010; Barratclough et al., 2014; Cassoff et al., 2011; Fowler, 1987; Wegner & Cartamil, 2012), and is often attributed to increased energy consumption associated with added drag from the gear (Feldkamp, 1985; Feldkamp et al., 1988; van der Hoop, Moore, Fahlman, et al., 2013). We used two separate approaches to determine the costs of entanglement: from measured blubber thicknesses at death, and from measured drag forces to estimate energetic demands for swimming. These methods are in close agreement: Differences in blubber thicknesses suggest that entangled right whales derive between 7.40 × 10^10^ J and 1.20 × 10^11^ J from blubber lipid catabolism over the duration of a lethal chronic entanglement, while physical propulsion models and drag measurements suggest fifteen right whales expended 3.95 × 10^9^ to 4.08 × 10^10^ J more energy over the time course of their entanglements.

From propulsive power requirements, we determined that survival beyond disentanglement was related to the additional amount of work whales performed while entangled: The amount of drag imposed by the entangling gear (and the resulting power required to overcome it) was not a predictor of the fate of entangled individuals; what mattered is the amount of time over which the additional energetic costs are incurred (*i.e*., work). This is intuitive, as health impacts are a significant predictor of entanglement survival (Robbins et al., 2015), and deterioration in health of entangled whales requires time (Barratclough et al., 2014; Cassoff et al., 2011; Rolland et al., 2016; Schick et al., 2013). We therefore established a threshold of 8.57 × 10^9^ J, the 0.75 quantile of the minimum additional work performed by whales that did not survive, with which to assess other entanglement cases to estimate the time course to death or the point at which energetic reserves may become compromised.

### 4.2 Entanglement in the context of life history

Large migratory animals undergo periods of fattening and fasting with remarkable adaptations for fat storage, mobilization, and utilization that allow them to meet demands in natural energy‐intensive periods. Our propulsive model suggests that drag from entanglement in fishing gear can incur energetic demands similar to costly life‐history stages. Daily additional costs of swimming while entangled (7.24 × 10^7^–7.52 × 10^8 ^J/day) are comparable to pregnancy (1.84 × 10^8^ J/day; Fortune et al., 2013) and migration (3.3 × 10^8^ J/day; van der Hoop, Moore, Fahlman, et al., 2013) and can be as high as costs of foraging (5 × 10^8^ J/day; McGregor, 2010) and reproduction (7.9 × 10^8^ J/day; Klansjcek et al., 2007; Figure 2a). Integrated over time, entanglements can cost 4.56(±5.30) × 10^9^ to 4.73(±7.95)×10^10^ J—as much as is energy expended to complete a migration (7.3 × 10^9^ J) and nearing what is required for an entire reproductive event (5.8 × 10^11^ J).

The timing of an additional energetic burden in the context of regular annual patterns, routines, and changes in energetic demands and variability cannot be ignored. We presented the relative and seasonally varying costs of life‐history events of large baleen whales, as well as the time of onset, cost, and duration of entanglements (Figure 2). The onset of these costs occurs in any month, when food may or may not be available, or when individuals may already be in negative energy balance, and last for many seasonal cycles (Figure 2c,d). Unlike migration or reproduction, where individuals undergo preparatory changes in body composition (e.g., Figure 3; Cherel, Robin, & Le Maho, 1988; Miller et al., 2011; Christiansen et al., 2013) and organ function (Weber, 2009), entanglement is unanticipated and is not necessarily associated with any particular seasonal cue; whether an individual animal has laid down fat reserves to cope with the energetic costs of entanglement is a matter of chance. Entanglement onset in the context of seasonal variability in body condition (e.g., Figure 3b) and annual variability in prey availability and quality only add to the complexity of how entangled individuals may or may not be able to sustain the energetic demands of drag loading.

### 4.3 Energetic costs and reproduction

The connection between increased drag, altered behavior, additional energy demand, high stress, and decreased breeding success has been established in studies addressing the effects of attaching scientific instruments (e.g., reviewed in Barron, Brawn, & Weatherhead, 2010); especially in birds and bats, added drag or weight from scientific instruments can lead to increases in energy expenditure, decreases in body condition, significant increases in corticosterones, and reduced nesting propensity and productivity (Barron et al., 2010; Elliott et al., 2012). Costs of transport significantly increase when instrumented (13.7%–100%; Gessaman & Nagy, 1988; Culik, Bannasch, & Wilson, 1994) even with devices designed to minimize increases in drag (17.4%; Culik et al., 1994) or weight. Our results contribute to recent literature to suggest that entangled whales may follow the same model of response to prolonged increases in drag of 52% on average: decreases in body condition (Barratclough et al., 2014; Cassoff et al., 2011; van der Hoop, Moore, Fahlman, et al., 2013; Moore et al., 2013), elevated stress hormones (Hunt, Rolland, Kraus, & Wasser, 2006), and reduced reproductive success (Knowlton, Hamilton, & Pettis, 2012).

For large whales, the most extreme variability in energetic demands and body condition are in females; body condition and blubber thicknesses increase significantly prior to pregnancy and are depleted through to the end of lactation (e.g., Christiansen, Dujon, Sprogis, Arnould, & Bejder, 2016; Lockyer, 1986; Miller et al., 2012; Williams et al., 2013). Models (Klansjcek et al., 2007; Villegas‐Amtmann, Schwarz, Sumich, & Costa, 2015) and observations (Lockyer, 1978, 1986, 1987; Williams et al., 2013) suggest plasticity in calving intervals depending on a female's nutritional demands and environmental conditions; small changes in energy availability have large impacts on calving interval and age at first parturition. Klansjcek et al. (2007) show that reducing energy acquisition of North Atlantic right whales by 16% inhibits reproduction and Villegas‐Amtmann et al. (2015) estimate that annual energetic losses of as little as 4% can limit calf production or survival in gray whales. We show that in close agreement with other large baleen whale species (Lockyer, 1981a,b, 1987; Villegas‐Amtmann et al., 2015), female right whales allocate 2% of their 4‐year reproductive cycle on pregnancy, 23% on lactation, and 75% on maintenance and recovery of energetic stores (Figure 5a,b). Entanglement can add 0.3%–7.8% to the 4‐year energy budget, increasing the time to energetic equilibrium or female recovery in preparation for the next pregnancy by months to years. The calculations of extended calving intervals we present do not include the time over which females were entangled—on average 99–621 (range 11–2459) days. Although there have been cases of right whales becoming entangled while pregnant and while lactating, it is likely that the time while entangled may also contribute to time between reproductive events, especially in species with spatiotemporally restricted breeding. The timing of entanglement during the reproductive cycle is another important consideration, where other capital breeders seem to be especially sensitive to energetic disturbance during pregnancy rather than during lactation (Villegas‐Amtmann et al., 2015).

Longer calving intervals (e.g., Figure 5a vs. b) suggest that reproduction is especially costly in balaenid whales (Knowlton et al., 1994; Nerini, Braham, Marquette, & Rugh, 1984), making resource allocation essential. Knowlton, Hamilton, & Pettis, et al. (2012) showed that reproductive female right whales with severe entanglement wounds have significantly longer calving intervals than females with no or minor wounds. Additionally, females carrying gear or with severe entanglement histories are significantly less likely to calve again. The energetic drain from the integrated additional power requirements over the time course of an entanglement can therefore have immediate or long‐term impacts on reproduction, critical to consider for both individual‐ and population‐level impacts.

During reproduction, the blubber thickness of the mother is reduced by around 4.5 cm (Miller et al., 2011). Our results show that blubber layers of entangled juveniles and adults at necropsy were 5.5 cm and 2.1 cm thinner, respectively, compared with individuals in the same age classes who died from non‐entanglement‐related causes (Figure 3a). Klansjcek et al. (2007) use the same ellipsoid method to estimate that a female right whale uses 5.8 × 10^11^ J of energy in a reproductive event, from becoming pregnant to carrying a calf through weaning; we have shown that right whales catabolize a similar magnitude of lipids during an entanglement.

Both the blubber thickness and propulsive model methods illustrate how the cost of entanglement over time can affect energy availability. Adequate energy reserves are a major contributor to reproductive success in many mammals (Frisch, 1984; Gittleman & Thompson, 1988; Lockyer, 1986; Young, 1976) including right whales (Miller et al., 2011, 2012). Baseline energy levels may be required to trigger ovulation: Numerous studies have related insufficient energy availability with skipping reproduction (Wasser & Barash, 1983) or prolonged periods of anoestrous (Wright, Rhind, Whyte, & Smith, 1992), and have identified threshold body conditions or blubber thicknesses beyond which the probability of pregnancy increases (Miller et al., 2011; Williams et al., 2013). On the 100‐point scale used for right whale health assessments, there appears to be a threshold condition of 67, below which reproduction does not occur; individuals in poor body condition are typically <60, including animals with entanglements (Rolland et al., 2016). The synergistic effects of metabolism, behavior, and chronic adrenal activation on stress‐induced anovulation have also been clarified (Berga & Loucks, 2007). While the focus is often on females, male reproductive development, interest, performance, and ability can also be affected by chronic or acute undernutrition and stress (Frisch, 1984). A significantly reduced androgen:estrogen ratio in a chronically entangled male right whale (EG 1102, Figure 2), coincident with the highest observed fecal glucocorticoid levels, suggests that stress‐related reproductive suppression can also occur in whales (Hunt et al., 2006).

### 4.4 Comparing the bioenergetic methods

The approaches taken here allow for independent estimates of energy expenditure related to a particular life‐history event. Changes in blubber thicknesses and estimated volumes only consider catabolism of lipid stored in the blubber layer, where the lipid content can vary with blubber depth and location along the body axis (Koopman et al., 2002; Parry, 1949). Entangled individuals may continue to forage or catabolize additional internal (e.g., muscular and visceral) lipid stores or protein sources, so changes in blubber thicknesses or volumes likely provide a conservative estimate of total energy use. However, the blubber volume method integrates all costs of living by including basal metabolic, stress, thermoregulatory, and health or repair costs that are currently not able to be measured. In addition, this method implicitly incorporates seasonal effects and energy requirements for swimming and overcoming entanglement‐related drag. These methods highlight the difference between normal and entangled whales instead of attempting to estimate total costs for either condition. While whole‐body bioenergetics models exist for cetaceans (Brodie, 1975; Kriete, 1995; New, Moretti, Hooker, Costa, & Simmons, 2013), including right whales (Fortune et al., 2013), it remains an unfortunate fact that the metabolic rates of large whales are unknown (e.g., see Gallivan, 1992); therefore, using whole‐body bioenergetic models to investigate the effects of entanglements is not possible.

Our method only incorporates measured differences in blubber thickness between entangled and nonentangled whales at death; it does not consider other changes in body girth in entangled whales, as there are few necropsy reports with entangled girth measurements and few instances of photogrammetry for entangled whales. Using normal body width minus a measured blubber thickness considers only changes in blubber volume, similar to Christiansen et al. (2013). The method excludes lipids metabolized from other body lipid depots (e.g., blood, viscera, muscle, bone). In most large whales, blubber contains majority of the total body adipose tissue (e.g., 96% in fin whales; Pond & Mattacks, 1988); however, whales may be metabolizing additional lipid depots or during the most extreme fasting conditions, directly catabolizing protein as an energy source (Aguilar, Giménez, Gómez‐Campos, Cardona, & Borrell, 2014; Cherel, Robin, Heitz, Calgari, & Le Maho, 1992; McCue, 2010; Rea, Rosen, & Trites, 2007; Worthy & Lavigne, 1987). Therefore, as it is based solely on lipid‐based energy sources in the blubber layer, this method represents an underestimate of metabolized energy.

The blubber model does not consider changes in the lipid density of the blubber. Blubber is a heterogeneous tissue, where density and lipid content can vary with blubber depth, location along the body, body length, and nutritive and ontogenetic condition (Aguilar & Borrell, 1990; Dunkin, McLellan, Blum, & Pabst, 2005; Dunkin et al., 2010; Koopman et al., 2002; Struntz et al., 2004). Significant changes in blubber lipid content can occur during seasonal fattening (Aguilar & Borrell, 1990; Lockyer, 1981a) or with reproductive status in balaenopterids (Lockyer, 1986, 1987). The rate at which blubber lipid content decreases with catabolism, and whether this decrease occurs in right whales as well, remains unknown and therefore could not be included in this model. However, if blubber lipid content is reduced in emaciated right whales as it is in emaciated dolphins (Dunkin et al., 2005), our estimates will remain conservative.

The power model reflects the cost of entanglement on top of the costs of normal existence, that is, for maintenance, thermoregulation, and routine activity. It therefore provides a simplified estimate that ignores the status (e.g., nutritional, health, or life history) of the animal at the time of entanglement. Dates of the actual entangling event are extremely difficult to determine; while survey effort can help determine the dates last seen gear‐free or first seen entangled, the time frame between these dates can vary from 3 to 1037 days for cases presented in this study (Figure 2). Whales are almost always in healthy body condition at the time last seen prior to entanglement (Robbins et al., 2015). The rate at which body shape and composition deteriorate will be a function of the drag and energetic requirements, as well as changes in the associated hydro‐ and thermodynamics that will contribute back to energetic costs. The dynamics of this feedback remain unquantified and are too complex to include here.

While the power method includes changes in swimming efficiency associated with entanglement (Aoki et al., 2011; Cornick, Inglis, Willis, & Horning, 2006; van der Hoop, Moore, Fahlman, et al., 2013; Nousek‐McGregor, Miller, Moore, & Nowacek, 2013; Williams, 1989), it assumes no change in swimming speed. Drag loading sometimes results in reduction in speed (Boyd, McCafferty, & Walker, 1997; Elliott, Davoren, & Gaston, 2007; van der Hoop et al., 2014; Lang & Daybell, 1963); however, there are instances where speed is maintained and additional energy consumption occurs (Williams, Friedl, & Haun, 1993). Few data exist on how entangled animals alter their swimming behaviors: Two right whales have been disentangled while wearing multisensor recording tags (DTAGs), from which ascent and descent speeds were resolved (van der Hoop, Moore, Fahlman, et al., 2013; van der Hoop et al., In Press ESR). While one individual (EG 3911) shows significantly slower vertical descent and ascent speeds (46% and 32% slower, respectively) when entangled compared with following disentanglement, the other individual (EG 4057) shows no difference in descent or ascent speeds between the two conditions (van der Hoop et al., In Press ESR). Satellite‐tag‐derived swimming speeds of entangled and nonentangled right whales (Baumgartner & Mate, 2005) do not indicate any difference in the average traveling speeds of these animals over the course resolution of ARGOS tags. Swimming speeds are often maintained in migratory species, where enough time must be left for essential activities such as feeding and reproduction (Weber, 2009). Our assumption that whales maintain speed likely provides an upper bound of power requirements.

## 5 Conclusions

Animals have adapted to prepare for, mitigate against, and recover from seasonal changes and natural fluctuations in energy demands and availability. We have shown that entanglement in fishing gear is an unpredictable event that can be extremely costly and last for days to years. Wingfield et al. (1998) proposed the “emergency life‐history stage” where unexpected events lead to redirection of behavior from normal life‐history stages, brought about by a suite of physiological and behavioral responses that can be sustained only for so long without lasting effects; entanglement in fishing gear can be considered the same. Even over the wide range of fishing gears, entanglement durations, and fates of individuals in our study, our results suggest that drag from entanglement can impact blubber stores and require energy investment on the order of magnitude as a reproductive event or migration. Recovery from such physiological stress and disturbance may limit an individual's future reproductive success, making entanglement a potential contributor to fluctuations in population growth. Historically, whale conservation measures have focused on reducing mortality; a shift is required to also address morbidity and the sublethal impacts on individuals and their reproductive rates.

## Data Accessibility

All data, figures, and tables are available on the Woods Hole Open Access Server (WHOAS), http://dx.doi.org/10.1575/1912/8048.

## Conflict of Interest

None declared.
