# Supplementary figures and images for: Entanglement is a costly life‐history stage in large whales
Source: Ecol Evol. 2016 Dec 11;7(1):92–106. doi: 10.1002/ece3.2615 (PMC5213775; doi:10.1002/ece3.2615)

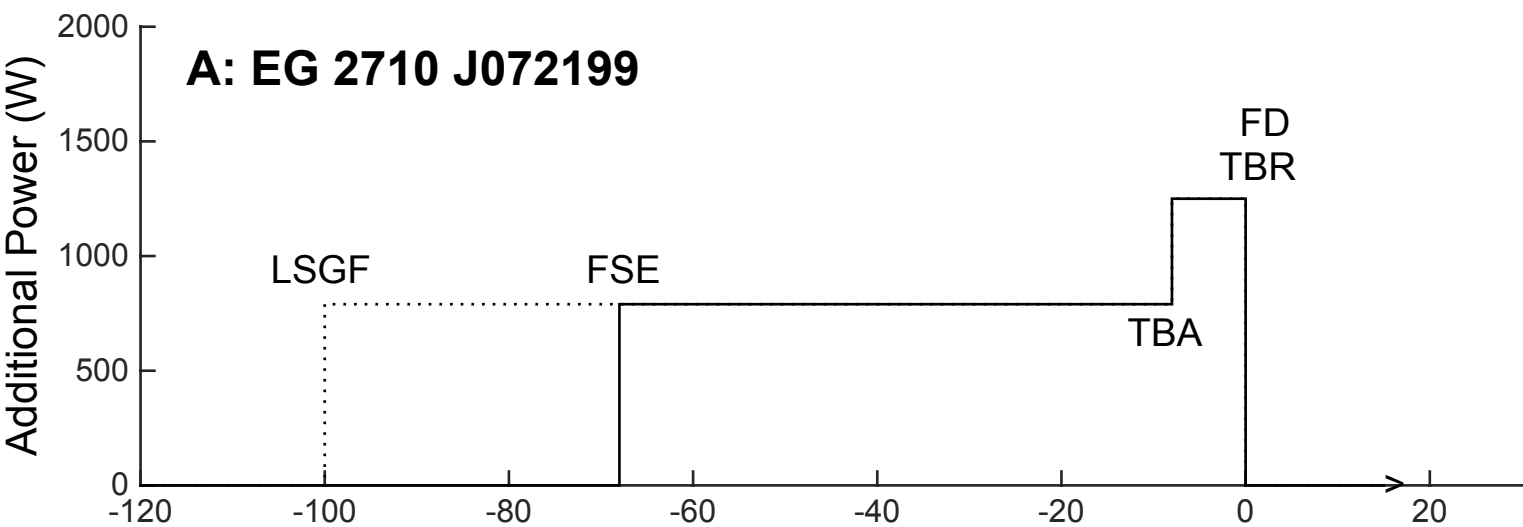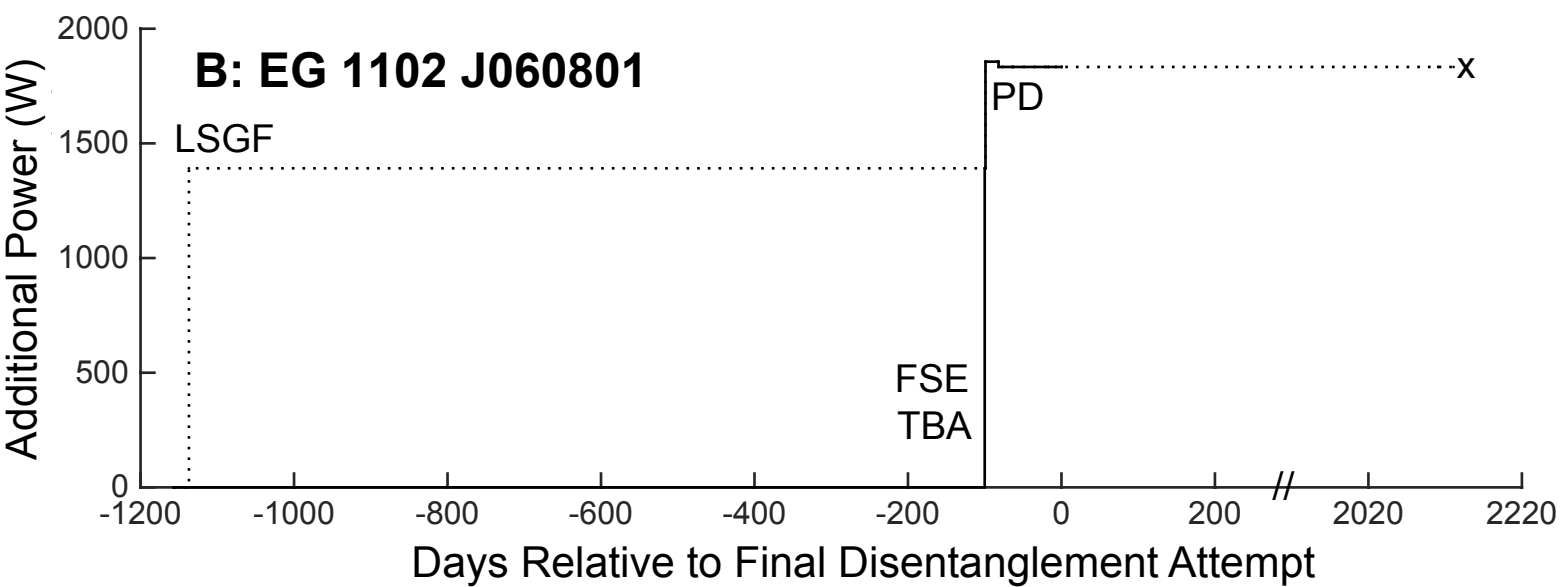

Supplement: Supplementary file 1 [file ECE3-7-92-s001.pdf]
